# Non-targeted and targeted metabolomics profiling of tea plants (*Camellia sinensis*) in response to its intercropping with Chinese chestnut

**DOI:** 10.1186/s12870-021-02841-w

**Published:** 2021-01-21

**Authors:** Tian Wu, Rui Zou, Dian Pu, Zengquan Lan, Bingyu Zhao

**Affiliations:** 1 Key Laboratory for Forest Resources Conservation and Utilization in the Southwest Mountains of China, Ministry of Education, Southwest Landscape Architecture Engineering Research Center of State Forestry Administration, Southwest Forestry University, Kunming, 650224 Yunnan China; 2 Ecology and Environment Department, Southwest Forestry University, Kunming, 650224 Yunnan China; 3 Southwest Institute of Ecology Development, Southwest Forestry University, Kunming, 650224 Yunnan China; 4 School of Plant and Environmental Sciences, Virginia Tech, Blacksburg, VA 24061 USA

**Keywords:** *Camellia sinensis*, Chinese chestnut, Amino acid, LC-MS, Agroforestry, Metabolic pathway

## Abstract

**Background:**

Intercropping is often used in the tea producing areas where land resources are not so abundant, and the produced green tea is tasted more delicious through a tea-Chinese chestnut intercropping system according to the experience of indigenous farmers. The length and weight of tea leaf increase under this intercropping system and their root systems are stratified vertically and coordinate symbiosis. However, the delicacy mechanism under the intercropping is not fully understood.

**Results:**

Green tea from the Chinese chestnut–tea intercropping system established in the 1980s ranked highest compared with a pure tea plantation from the same region. Based on the non-targeted metabolomics, 100 differential metabolites were upregulated in the tea leaves from intercropping system relative to monoculture system. Twenty-one amino acids were upregulated and three downregulated in response to the intercropping based on the targeted metabolomics; half of the upregulated amino acids had positive effects on the tea taste. Levels of allantoic acid, sugars, sugar alcohols, and oleic acid were higher and less bitter flavonoids in the intercropping system than those in monoculture system. The upregulated metabolites could promote the quality of tea and its health-beneficial health effects. Flavone and flavonol biosynthesis and phenylalanine metabolism showed the greatest difference. Numerous pathways associated with amino acid metabolism altered, suggesting that the intercropping of Chinese chestnut–tea could greatly influence amino acid metabolism in tea plants.

**Conclusions:**

These results enhance our understanding of the metabolic mechanisms by which tea quality is improved in the Chinese chestnut–tea intercropping system and demonstrate that there is great potential to improve tea quality at the metabolomic level by adopting such an intercropping system.

**Supplementary Information:**

The online version contains supplementary material available at 10.1186/s12870-021-02841-w.

## Background

Tea [*Camellia sinensis* (L.) O. Kuntze] is a vital crop consumed worldwide, primarily in the form of a beverage processed by fresh leaves in different ways [1]. Tea originated as an understory plant of the tropical rainforest and therefore, it prefers warm, moist, shaded conditions with diffuse light and acidic soils [2]. Given these preferences, agroforestry intercropping systems offer a convenient strategy for tea cultivation and conservation. Agroforestry intercropping is an ancient and effective planting method in which one plant species is grown alongside another, thereby increasing yields and economic returns, reducing weeds and pests, improving light interception and utilization, and increasing iron nutrition, among other benefits [3–6]. Agroforestry intercropping has been commonly practiced in many parts of the world where arable land is scarce and hunger remains [7, 8].

The strategy of interplanting tea with other tree species has been developed in various forms and applied in different tea-producing regions. Some tall tree species are used as shade trees in tea plantations, including coconut (*Cocos nucifera*), orange (*Citrus reticulata*), waxberry (*Myrica rubra*), loquat (*Eriobotrya japonica*), and gingko (*Ginkgo biloba*) [9]. Because of the decreased use of pesticides in intercropping tea plantations, the resulting tea leaves are likely to have less chemical contamination problems [9]. Intercropping of rubber and tea to ameliorate land degradation has been practiced in mountain areas susceptible to soil erosion. For example, intercropped cultivation of rubber and tea plant was recommended to some provinces of China in the 1970s, which resulted in positive results on tea quality and soil and water conservation and tea quality [10].

Chinese chestnut (*Castanea mollissima* Blume) and tea plants are classic intercropping partners, and researchers have studied some different aspects of the Chinese chestnut–tea intercropping system. Chinese chestnut is one of the leading economically important species in the chestnut species [11]. As practiced in northern China, chestnut–tea intercropping reduces the intensity of illumination on tea plants, lowering air and soil temperatures around the plants, thereby increasing air humidity. This altered microclimate can improve tea quality and yield [12]. The intercropping system allows the root systems of Chinese chestnut and tea plants to be stratified vertically and coordinate the symbiosis, which increases the dry weight of tea plants’ root system [13]. In addition, the improved soil structure in the intercropping system also results in increased plant leaf length and weight [14]. Therefore, intercropping Chinese chestnut and tea not only improve tea production but also has ecological benefits, representing a sustainable means of tea plantation management [15].

Baohong tea [*Camellia sinensis* (L.) O. Kuntze cv. Baohong] is the only small-leaf tea grown in Yunnan province, China. It is usually made into a green tea with a unique natural floral aroma; this high-aroma green tea is considered to be almost comparable to the Longjing tea of Hangzhou, China. Since 1965, Baohong tea plants were cultivated over nearly 40 ha on Baohong Mountain in Yiliang county, Yunnan Province. However, in that era of relatively low economic and social development, the income from selling Baohong tea alone was relatively low. Chinese chestnut is also a traditional woody crop in the local area. Therefore, a group of Chinese chestnut trees was also planted in approximately 10 ha of the Baohong tea plantation in the 1970s. In Yiliang, Chinese chestnuts are harvested beginning at about 5 years after cultivation, the market for Chinese chestnut was strong, and the presence of chestnut did not affect the growth of the tea plants. The doubled income from a single piece of land increased tea farmers’ profitability, so another group of Chinese chestnuts was added to the Baohong tea plantation (~ 10 ha) in the 1980s and 1990s, respectively. In the late 1990s, as the worldwide tea consumption increased, pure tea plantations also become profitable, and the Baohong tea plantation did not plant additional trees on its remaining few hectares. Irrigation and fertilization of tea plants on Baohong Mountain are systematically managed, and the plants were trimmed once a year to ensure that their height was maintained at approximately 1 m. After decades of development, these cultivation systems, including both the pure tea system and the Chinese chestnut–tea intercropping system, have been preserved till the present day, becoming a model for Chinese chestnut–tea intercropping around Kunming and even across China. Although the ecological and economic benefits of Chinese chestnut–tea intercropping have been clearly demonstrated in the Baohong tea plantations, however, it is unclear whether intercropping leads to improved tea quality, as fresh tea leaves from all the Baohong production systems have traditionally been picked and mixed together.

The tea quality is assessed based on the content of characteristic sensory substances, including amino acids, organic acids, sugars, lipids, as well as some bioactive substances like phenolics [16]. Amino acids contribute to the flavor of green tea and confer some health benefits, such as anti-inflammatory, anti-microbial, and positive neurological effects [17]. Organic acids, which also have great health benefits, can affect tea quality due to the taste and their biological activities [16]. Free sugars are essential for catechin synthesis, which can help the formation of tea flavors during processing [16]. Various forms of lipid present in tea leaf that can be altered by heat or oxidation during processing, can generate volatile compounds and result in the characteristic taste of green tea [18]. Therefore, effectively and accurately characterizing the dynamics of these complicated chemical substances in tea leaf is required for tea quality assessment. Thus far, a few research have demonstrated that the chestnut–tea intercropping system could improve tea quality by the measuring of one class or a small number of chemical substances in the tea leaf, which cannot fully reflect the complexities of tea quality.

Metabolomics technology provides a more comprehensive approach for measuring the complicated chemical components in tea [19]. Thus far, the effects of Chinese chestnut–tea intercropping on the metabolomics profiling of fresh tea have not been investigated. Systemic characterization of tea composition could be lay the foundation for further improve the quality of green tea through agronomic practice and tea plantation management. Hence, we investigated the effects of Chinese chestnut–tea intercropping systems on tea quality by performing sensory evaluation and measurements of major green tea components and by documenting differences in green tea metabolites through targeted and non-targeted metabolomics with LC-MS. We identify more than 100 differential metabolites were upregulated in the tea leaves from intercropping system relative to monoculture system. Pathway analysis suggests the lavone and flavonol biosynthesis and phenylalanine metabolism, and amino acid metabolism are significantly altered, suggesting that the intercropping of Chinese chestnut–tea could greatly influence allantoic acid, sugars, sugar alcohols, and oleic acids, and amino acid metabolism in tea plants, which are are positively related to improved tea taste. Our results supply a theoretical foundation for developing the ecological model of intercropping with Chinese chestnut and even with other tree species.

## Methods

### Plant materials and growth conditions

The Baohong tea plants were planted in the 1960s on the Baohong mountain, belonging to Baohong Tea Industry Co. LTD, China. The mountain is located at an average elevation of about 1900 m and has a northern semi-tropical monsoon climate with an average atmospheric temperature of 16.3 °C and average humidity of 80%. The sampling was conducted on private land, and we confirm that the land owner gave permission for this. We collected four groups, three from tea plants in the chestnut–tea intercropping system planted in the 1970s, 1980s, and 1990s, and one from the pure tea plantation, which served as the control (Table S1). The groups were designated 70 T, 80 T, 90 T and T. A bud and two leaves were carefully collected from a tea plant by hand on March 24, 2019. The tea plant covered by the chestnut crown was sampled in the chestnut–tea intercropping system. We took 150 plants in total, dividing them into groups of 50 plants and regarding each group as a biological replicate, and then picked 5 to 10 buds and leaves from each plant. This was done completely at random. This was done completely at random. For each biological replicate of each group, we divided the leaves into two portions. For each biological replicate, all the leaves from 50 plants were placed in a same bamboo bag and then divided into two portions. One portion was placed in a sampling tube, then quick-frozen and temporarily stored in liquid nitrogen, taken to the laboratory, and stored in a freezer at − 80 °C for targeted and non-targeted metabolomics measurements. The other portion was taken to the Baohong tea processing workshop, where it was steamed in an electric steamer for 4 min. After steaming, the tea leaves were thinly spread on a bamboo mat and dried under the sunshine outdoors for about 4 h. It was performed from 1 to 5 p.m. of March 24, with the temperature from 25 to 23 °C, the wind of SW 12.3 ~ 17.7 mph, and the solar radiation 682 kJ/m^2^. The dried tea leaves were sealed in a self-sealing bag for sensory evaluation and measurement of caffeine, tea polyphenols and free amino acids.

### Sensory evaluation of tea samples

Tea sensory evaluation was carried out by the National Standards of the People’s Republic of China (GB/T 23776–2018) via a trained panel of five senior tea-tasters. It was performed to establish preference rating for appearance, the color of tea liquid, scent, taste, and infused leaf, and be graded according to 20, 20, 25, 25, and 10 score respectively, which means the higher the evaluation, the higher the score. Each sub-item was graded separately, and they were summed up at last as the final score. The blind evaluation system was adopted: that is, the tea-tasters knew nothing about what each tea sample was and could only get the tea sample number. They were supplied with a prescribed questionnaire to document their sensory evaluations. The sensory evaluation was conducted in a room with 25 °C and a humidity of 67%.

### Determination of caffeine content, tea polyphenols and free amino acids

Caffeine was determined by the National Standards of the People’s Republic of China (GB/T 8312–2013) using HPLC (High-Performance Liquid Chromatography). The standard curve was drawn as y = 32.064x-8.2625 (*R*^*2*^ = 0.9999). A part of the solution was filtered through the filter membrane of 0.45 μm, and the liquid was accurately measured and injected into the HPLC and compared with the standard curve.

The determination of tea polyphenols was conducted by the National Standards of the People’s Republic of China (GB/T 8313–2008). According to the absorbance and concentration of the gallic acid working solution, the standard curve y = 0.005x + 0.0728 (*R*^*2*^ = 0.997) was drawn. Absorption was determined at 765 nm using Ultraviolet-visible spectroscopy (UV-2401, Shimadzu).

Amino acids were detected by the method of the National Standards of the People’s Republic of China (GB/T 8314–2013). A standard curve was drawn according to the absorbance and the concentration of theanine, which was y = 2.7841x-0.1534 (*R*^*2*^ = 0.9929). The absorbance of the sample was determined using the reagent solution as blank control, and the amino acid content was obtained by comparison with the standard curve.

### Non-targeted metabolomics

The non-targeted metabolomics method was performed following the procedure as reported by Zeng et al. [20]. Briefly, metabolites were extracted for the UHPLC-QTOF-MS analysis. LC-MS/MS analyses were conducted by UHPLC system (1290, Agilent Technologies) with a UPLC BEH Amide column (1.7 μm 2.1*100 mm, Waters) coupled to TripleTOF 6600 (Q-TOF, AB Sciex). MS unprocessed data files were transformed to the mzXML format by ProteoWizard, and processed by R package XCMS (version 3.2). The preprocessing results created a data matrix containing the retention time (RT), mass-to-charge ratio (m/z) values, and peak intensity. R package CAMERA was applied for peak annotation after XCMS data processing. A second mass spectrometer (MS2) database was used in metabolites identification. The metabolites with the qualitative analysis with MS2 were selected, which were discovered depending on the metabolome databases of HMDB, PubChem, KEGG and METLIN.

### Targeted metabolomics

The analytical methods of targeted amino acids mainly referred to the instruction by Zhu et al. [21]. The UHPLC separation was performed by Agilent 1290 Infinity II series UHPLC System (Agilent Technologies), furnished with Waters ACQUITY UPLC BEH Amide column (100 × 2.1 mm, 1.7 μm). The temperature of the column and auto-sampler was set respectively at 35 °C and 4 °C. An Agilent 6460 triple quadrupole mass spectrometer (Agilent Technologies) with AJS electrospray ionization (AJS-ESI) interface, was used as assay development. MRM data were collected and processed by Agilent Mass Hunter Work Station Software (B.08.00, Agilent Technologies). The level was eliminated from the calibration if S/N (signal-to-noise ratio) was less than or equal to 20 or accuracy of the calibration was beyond 80–120%.

### Statistical analyses

In order to achieve an outline of the figures of chemical variation in the 80 T and T samples, the significant differences were investigated by PCA pattern recognition with SIMCA software (V14.1, Sartorius Stedim Data Analytics AB, Umea, Sweden). To get a higher level of group separation and a better understanding of T and 80 T samples liable for categorization, supervised OPLS-DA were used. Metabolites (VIP > 1.0) were supposed as vital metabolites for potential discrimination of samples in the OPLS-DA models. We used the Student’s test, which is commonly used for metabolomics statistical analysis. *P* < 0.05 in the Student’s test was supposed statistically significant.

## Results

### Green tea from the intercropping of chestnut cultured in the 1980s ranked highest in sensory evaluation

80 T received the highest sensory evaluation among the four green tea samples. The taste scores of 70 T, 80 T and 90 T were 88.5, 91 and 81.5, respectively (Table S2). 80 T was fresh, exhibited a strong aroma, and had the best taste. 70 T was fresh and tender and had an aroma. The taste of T sample was described by the tea-taster as pure and normal with a clean flavor, and its score was 82.5. These results indicated that Chinese chestnut–tea intercropping had a positive impact on the taste and aroma of Baohong green tea, especially for the tea grown with Chinese chestnuts planted in the 1980s.

### Green tea from the intercropping of chestnut cultured in the 1980s had the lowest polyphenol:amino acid ratio

**Fig. 1:**
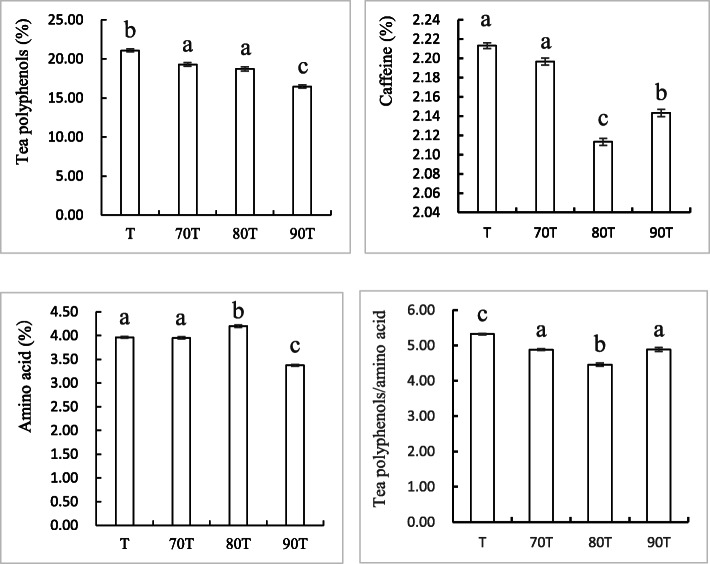
Detection of major components of steamed green tea of T, 70 T, 80 T and 90 T

Amino acid content is higher and polyphenol content is lower in Baohong green tea from the intercropping system, leading to a lower ratio of tea polyphenols to amino acids, particularly in the 80 T sample. This low ratio is often considered to be a good indicator of green tea quality [22]. This result is consistent with the sensory evaluation results, which indicated that 80 T green tea had the best taste. As shown in Fig. 1, the tea polyphenol contents of 70 T, 80 T and 90 T were 19.28, 18.73 and 16.48%, respectively, and lower than that of the T control sample (21.09%). Likewise, their caffeine contents were 2.19, 2.12 and 2.15%, again lower than that of the T sample (2.22%).

The 80 T sample was selected as the intercropping sample for the subsequent metabolomics analysis compared with the monoculture sample because 80 T sample received the highest sensory evaluation and had the most appropriate levels of major tea chemical components.

### Metabolite profiles differed under the intercropping vs. monoculture

**Fig. 2:**
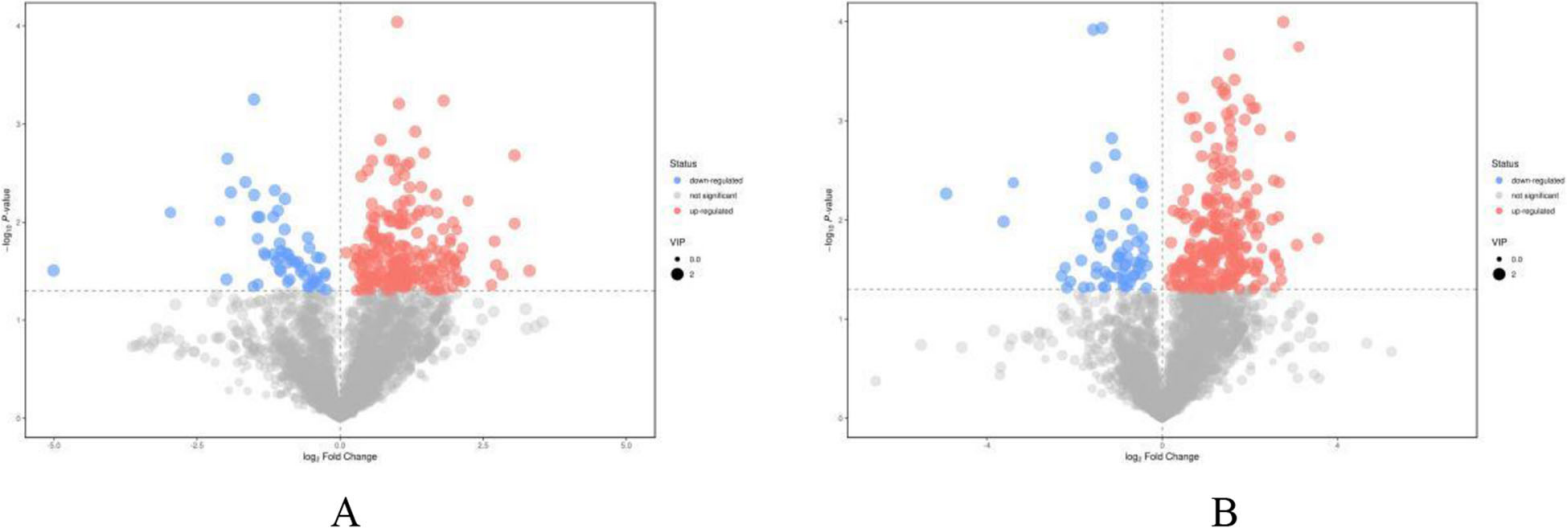
The volcano plots of positive (**a**) and negative (**b**) modes based on the non-target metabolomics in the tea samples of 80 T (intercropping) vs. T (monoculture)

Figure 2 presents base peak intensity (BPI) chromatograms in positive and negative ionization modes for the samples under the intercropping vs. monoculture. Metabolites differed significantly between samples from the intercropping system and those from the pure tea system. In total, 3450 and 3538 metabolite ion features differed from the two culture systems in the two modes (Figure S1), respectively. A volcano plot (Fig. 2) showed that there were more upregulated than downregulated metabolites under the intercropping vs. monoculture, suggesting the intercropping not only caused differences in metabolites but also promoted metabolite accumulation.

**Fig. 3:**
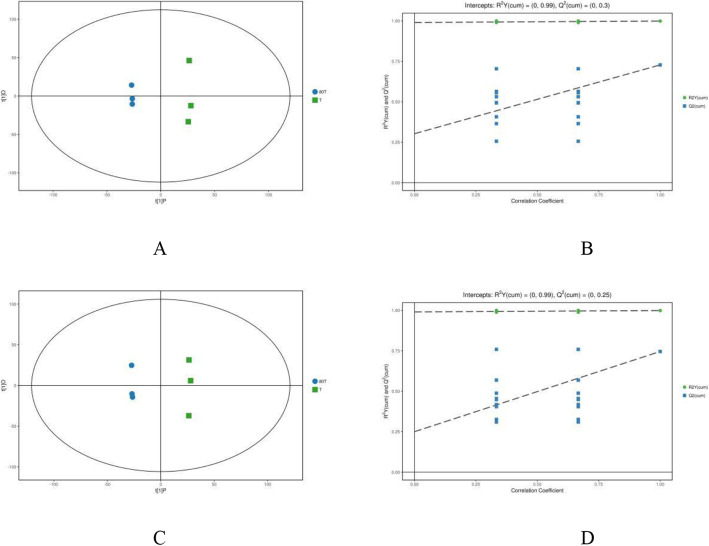
OPLS-DA models based on the non-target metabolomics for the data from positive (**a**, **b**) and negative (**c**, **d**) ionization modes for the tea samples of 80 T (intercropping) vs. T (monoculture): score plots (**a**, **c**) and permutation plots (**b**, **d**)

OPLS-DA models all showed a great goodness-of-fit (*R*^2^X) and high predictability (*Q*^2^), with 0.478 and 0.728 in positive mode (Fig. 3a) and 0.445 and 0.746 in negative mode (Fig. 3c) for the samples under the intercropping vs. monoculture. The OPLS-DA models were confirmed by response permutation testing (RPT), disclosing the absence of overfitting (Fig. 3b and d) and discovered no false positives in our data. PCA score plots indicated that the metabolic profiles of tea leaves under the intercropping vs. monoculture differed markedly. Four-component PCA score plots were established for the data from both positive modes (*R*^2^X = 0.99, *Q*^2^ = 0.3) and negative modes (*R*^2^X = 0.99, *Q*^2^ = 0.25). All samples were in the 95% confidence interval (Hotelling’s *t*-squared ellipse).

### Numerous metabolites with MS2 identifications differed under the intercropping vs. monoculture

**Fig. 4:**
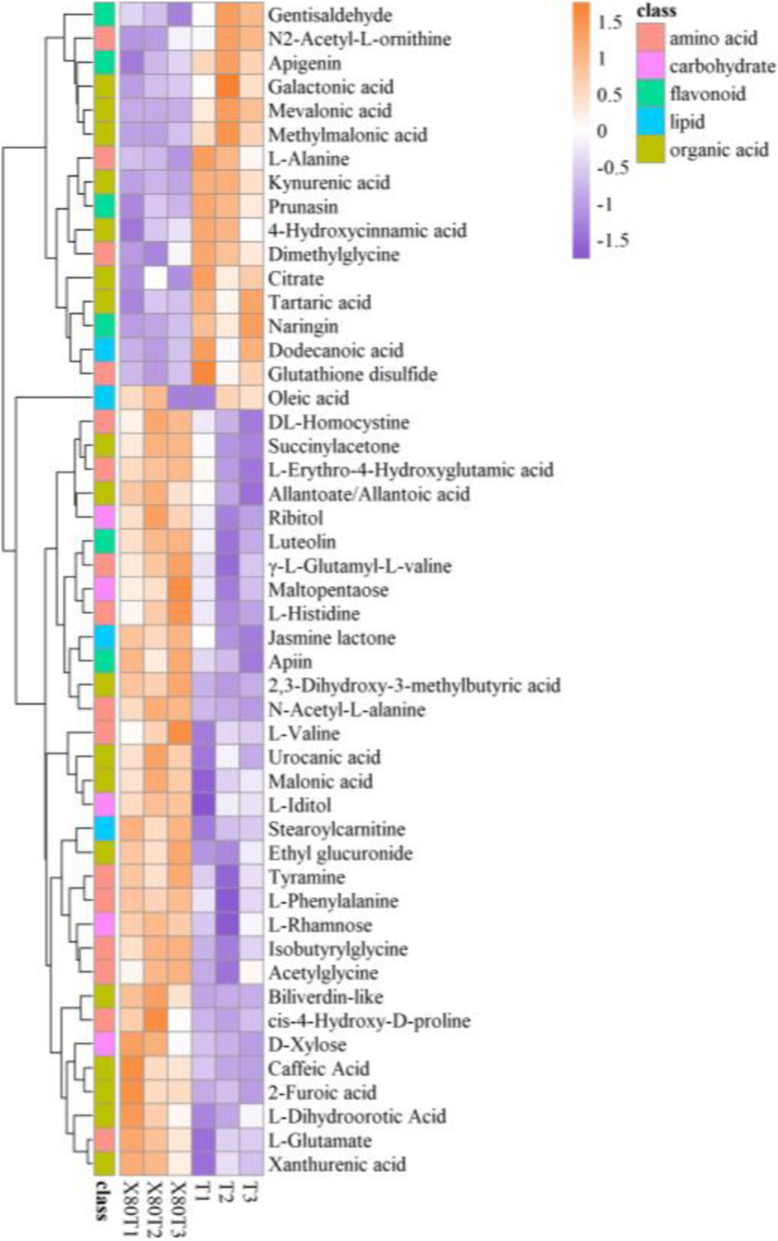
Heat map showing the differential metabolites with MS2 based on the non-target metabolomics in the tea samples of 80 T (intercropping) vs. T (monoculture)

A total of 100 metabolites with MS2 identifications, 65 in positive mode and 35 in negative mode, differed significantly under the intercropping vs. monoculture (VIP>1.0 and *P*<0.05) depending on the HMDB, PubChem, KEGG and METLIN metabolome databases. The differential metabolites could be sorted as amino acids, organic acids, carbohydrates, lipids and flavonoids (Table 1). These are flavor compounds, bioactive components, or compounds that release volatiles during tea manufacturing and are considered to be aroma precursors [23]. They are the most important basic materials in fresh tea leaves that determine tea quality. Heat maps were constructed to acquire an obvious outline of metabolites between the samples (Fig. 4). The differential metabolites could clearly be divided into up- and down-regulated groups, and the majority (68%) were upregulated.

**Table 1:** Differential metabolites with MS2 based on the non-target metabolomics in the tea samples of 80 T (intercropping) vs. T (monoculture). 1A Metabolites in positive modes. 1B Metabolites in negative modes

Classification	MS2 name	RT	VIP	***P*** value	Fold change
1A					
Amino acids	L-Valine	338.138	1.12	0.05	3.59
	L-Phenylalanine	239.997	1.72	0.03	1.19
	cis-4-Hydroxy-D-proline	340.546	1.77	0.03	1.30
	Acetylglycine	356.8465	1.17	0.05	3.17
	Isobutyrylglycine	49.179	1.24	0.01	4.70
	DL-Homocystine	298.929	1.64	0.02	2.39
	L-Erythro-4-Hydroxyglutamic acid	394.8715	1.59	0.03	2.20
	Tyramine	239.973	1.75	0.02	1.23
	Theanine	300.140	0.70	0.54	1.06
	N2-Acetyl-L-ornithine	340.1385	1.68	0.04	0.74
	Dimethylglycine	314.045	1.74	0.02	0.75
	Glutathione disulfide	475.8755	1.80	0.03	0.60
	L-Alanine	365.347	1.85	0.01	0.68
Organic acids	Urocanic acid	311.8865	1.69	0.02	1.56
	Xanthurenic acid	206.8875	1.65	0.02	1.92
	Allantoate/Allantoic acid	343.072	1.22	0.04	3.13
	Biliverdin	111.658	1.97	0.02	2.81
	Mevalonic acid	322.165	1.99	0.03	0.03
	Kynurenic acid	172.561	1.88	0.01	0.44
Lipids	Jasmine lactone	277.9675	1.76	0.02	1.21
	Stearoylcarnitine	139.053	1.85	0.01	1.47
	Dodecanoic acid	77.1265	1.75	0.02	0.79
Carbohydrates	L-Rhamnose	316.302	1.22	0.03	2.70
	Maltopentaose	493.523	1.67	0.05	1.30
	D-Xylose	56.531	1.79	0.02	1.49
	Ribitol	163.045	1.78	0.02	1.33
	L-Iditol	114.379	1.53	0.04	1.40
Flavonoids	Apiin	331.713	1.76	0.01	1.64
	Luteolin	327.191	1.53	0.02	3.03
	Apigenin	181.574	1.16	0.01	0.23
	Naringin	175.755	1.90	0.01	0.51
	Prunasin	52.481	1.86	0.01	0.47
	Gentisaldehyde	323.015	1.73	0.03	0.62
1B					
Amino acids	N-Acetyl-L-alanine	251.6825	1.97	0.00	2.13
	L-Glutamate	407.621	1.78	0.02	1.15
	L-Histidine	208.502	1.57	0.03	4.33
	gamma-L-Glutamyl-L-valine	351.7275	1.71	0.03	1.61
	Theanine	298.777	0.94	0.40	1.48
Organic acids	Malonic acid	374.182	1.60	0.03	1.59
	Succinylacetone	227.898	1.71	0.02	2.00
	Ethyl glucuronide	335.646	1.81	0.01	1.68
	2-Furoic acid	285.4685	1.88	0.01	1.45
	2,3-Dihydroxy-3-methylbutyric acid	147.616	1.98	0.00	3.95
	Caffeic Acid	327.572	1.88	0.02	2.68
	L-Dihydroorotic Acid	143.736	1.63	0.05	2.11
	Galactonic acid	353.881	1.76	0.05	0.57
	Tartaric acid	398.58	1.79	0.02	0.67
	4-Hydroxycinnamic acid	145.027	1.56	0.04	0.44
	Citric acid	448.29	1.68	0.04	0.71
	Methylmalonic acid	445.2185	1.92	0.01	0.73
	Kynurenic acid	172.775	1.98	0.00	0.45
Lipids	Oleic acid	40.499	1.97	0.00	1.68

To better understand the relationship between tea metabolites and tea quality in the intercropping system, we discussed the major classes of differential metabolites in detail below.

### Amino acids

Eleven upregulated and four downregulated amino acids were identified under the intercropping vs. monoculture based on non-targeted metabolomics. Interestingly, theanine was upregulated in two modes, [1.06-fold in positive mode (VIP = 0.69 and *P* = 0.54) and 1.48-fold in negative mode (VIP = 0.94 and *P* = 0.40)], although it did not satisfy the statistical conditions of VIP>1.0 and *P*<0.05. Theanine is the most abundant and important in tea and primarily responsible for its umami taste [24]. Differences in theanine were not statistically significant, suggesting that the taste improvement associated with intercropping was not dependent on theanine. The role of theanine in taste perception is not denied here, but it only showed there were minor differences in theanine content between the intercropping system and the pure tea plantation.

To verify the contents of free amino acids—and also because of the vital role of free amino acids to the savory taste of tea—we further performed an in-depth analysis of free amino acids using targeted metabolomics on the same samples used for non-targeted metabolomics.

**Fig. 5:**
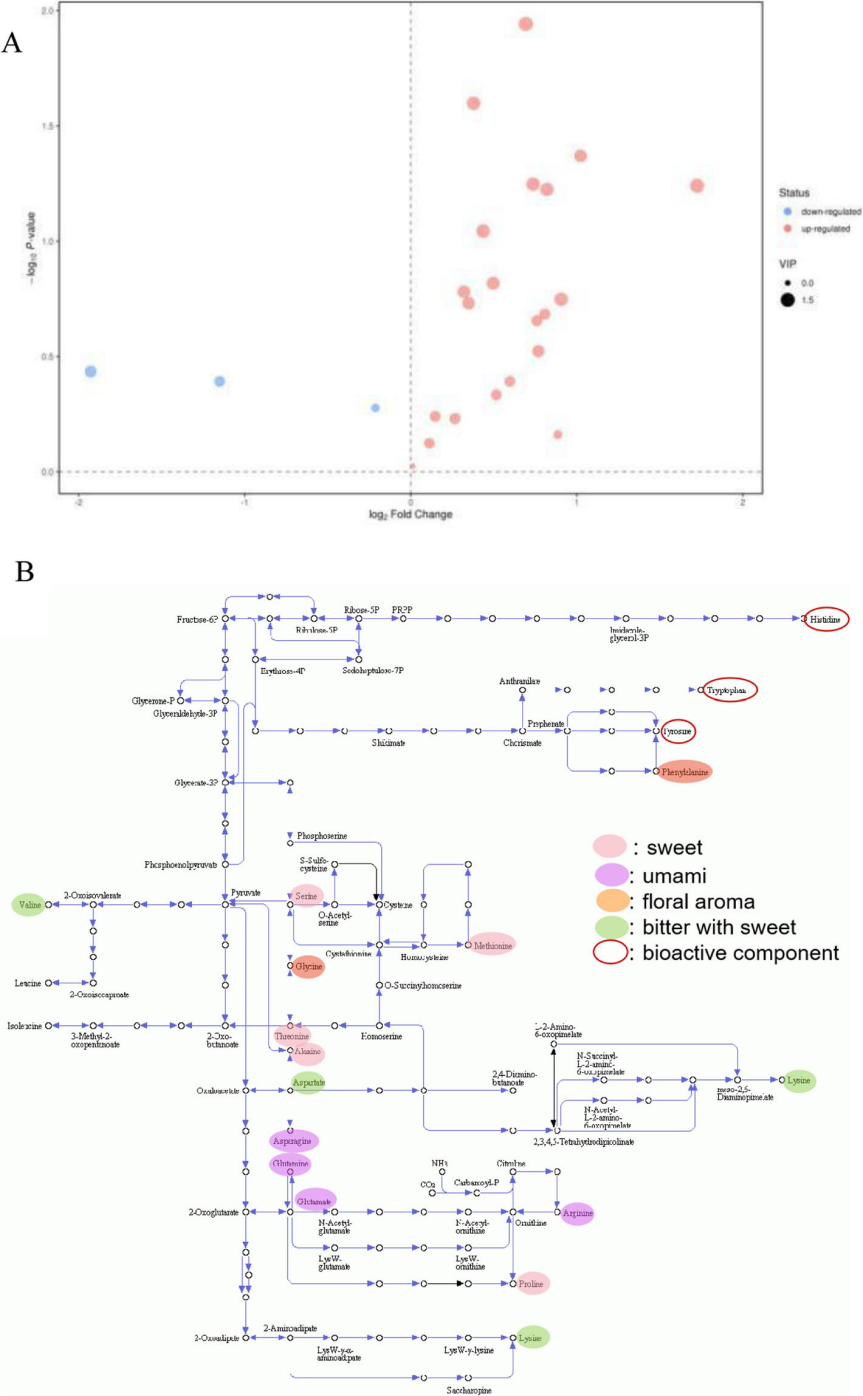
Volcano plot (**a**) and the up-regulated amino acids on the metabolomics in biosynthesis of amino acids (map01230) (**b**) for 80 T (intercropping) vs. T (monoculture) based on the target metabolomics

Targeted metabolomics identified 21 upregulated and three downregulated amino acids under the intercropping vs. monoculture showed in Table 2 and Fig. 5a. GABA (γ-aminobutyric acid) which is a nonprotein amino acid and an important bioactive tea component that provides various health benefits [25], was upregulated 1.62-fold in samples of the intercropping vs. monoculture. Levels of L-alanine (Ala), L-proline (Pro), Phe, L-serine (Ser), and L-glutamine (Gln) were markedly higher under the intercropping vs. monoculture.

**Table 2:** The amino acids based on the target metabolomics in the tea samples of 80 T (intercropping) vs. T (monoculture)

Name	Abbreviation	MEAN 80 T (nmol/g)	MEAN T (nmol/g)	VIP	***P*** value	Fold change	Log_Fold change	Flavor or not
L-Methionine	Met	77.24	60.68	1.07	0.19	1.27	0.35	sweet
L-Proline	Pro	175.52	124.45	1.19	0.15	1.41	0.50	sweet, bitter
L-Alanine	Ala	1786.97	1375.01	1.31	0.03	1.30	0.38	sweet
L-Threonine	Thr	4539.63	3356.81	1.31	0.09	1.35	0.44	sweet
L-Serine	Ser	13,202.69	7802.77	0.73	0.22	1.69	0.76	sweet
L-Asparagine	Asn	3532.84	1069.28	1.49	0.06	3.30	1.72	umami
L-Glutamic acid	Glu	18,283.39	12,094.54	0.66	0.41	1.51	0.60	umami
L-Aspartic acid	Asp	12,427.77	8698.41	0.64	0.46	1.43	0.51	umami
L-Glutamine	Gln	17,306.94	9891.39	0.75	0.21	1.75	0.81	umami
L-Phenylalanine	Phe	293.31	291.15	0.21	0.95	1.01	0.01	floral aroma
L-Glycine	Gly	777.64	623.06	1.11	0.17	1.25	0.32	floral aroma
L-Tryptophan	Trp	851.86	769.41	0.70	0.57	1.11	0.15	bitter
L-Arginine	Arg	3785.44	3147.93	0.74	0.59	1.20	0.27	bitter, sweet
L-Histidine	His	2696.35	1439.46	1.33	0.18	1.87	0.91	bitter
L-Valine	Val	1319.02	1220.96	0.61	0.75	1.08	0.11	bitter, sweet
L-Tyrosine	Tyr	6.37	3.61	1.29	0.06	1.76	0.82	bitter
L-Lysine	Lys	783.85	470.56	1.30	0.06	1.67	0.74	bitter, sweet
4-Aminobutyric acid	GABA	1380.00	854.07	1.54	0.01	1.62	0.69	no
L-Citrulline	Cit	314.20	184.52	1.00	0.30	1.70	0.77	no
β-Alanine	β-Ala	15.95	7.85	1.07	0.04	2.03	1.02	no
L-Ornithine	Orn	320.31	173.38	0.39	0.69	1.85	0.89	no
4-Hydroxyproline	4-Hyd	56.33	214.33	0.90	0.37	0.26	−1.93	no
1-Methyl-L-histidine	1-Mhis	107.07	237.82	0.67	0.41	0.45	−1.15	no
3-Methyl-L-histidine	3-Mhis	115.18	133.47	0.35	0.53	0.86	−0.21	no

Half of the upregulated amino acids had documented roles in improving the flavor of green tea, whereas none of the downregulated amino acids were known to influence tea flavor. Among the upregulated flavor-promoting amino acids, L-methionine (Met), Pro, Ala, L-threonine (Thr), and Ser are sweet-tasting compounds; Glu, Gln, L-asparagine (Asn), and L-aspartic acid (Asp) contribute an umami taste; and Phe and L-glycine (Gly) produce a floral aroma [reviewed in Yu and Yang] [18]. Asn showed the greatest upregulation in the intercropping (3.30-fold). It is worth noting that amino acids that make the greatest contribution to taste, such as Glu, Asp and Gln, were present at concentrations above 10,000 nmol/g in the intercropping and therefore provided rich raw materials for producing green tea with high quality in the intercropping system.

To display the upregulated flavor-promoting and bioactive amino acids more intuitively, they are labeled with ovals in Fig. 5b and color-coded according to their properties: sweet in pink, umami in orchid, floral aroma in orange, and bitter and sweet in green. Bioactive amino acids are indicated with a dark red ring, based on the KEGG pathway ‘biosynthesis of amino acids’ (map01230). The abundant upregulated amino acids in the intercropping suggested that fresh leaves from the intercropping system were more suitable for the production of green tea.

Amino acids downregulated in the intercropping vs. monoculture included 4-hydroxyproline, 1-methyl-L-histidine (1-MHis) and 3-methyl-L-histidine (3-MHis). The recognition threshold for sweetness is lower in 4-hydroxyproline than in other sweet amino acids such as Pro, Ala, and Thr [26]. 1-MHis and 3-MHis have not been reported as taste compounds. Therefore, we speculated that its downregulation in the intercropping may have had little effect on tea sweetness, especially given the upregulation of other sweet amino acids.

### Organic acids

We detected 11 significantly upregulated and seven significantly downregulated organic acids in samples under the intercropping vs. monoculture. The greatest upregulation (3.3-fold) was observed in allantoic acid, the conjugate acid of allantoate. Allantoin and allantoate exist in the caffeine degradation of caffeine-containing plants; specifically, caffeine is converted to allantoic acid by way of theophylline, 3-methylxantine, xanthine and uric acid [27, 28]. The relative content of allantoin in plant may reflect the caffeine degradation [29]. Therefore, we speculate that caffeine may have been degraded into allantoic acid in the intercropping system, causing accumulation of allantoic acid, consistent with the lower caffeine content measured in the intercropping (Fig. 1).

Xanthurenate (XA) increased and kynurenic acid (KA) decreased in the intercropping samples relative to the T samples in two modes. XA and KA are both components of the kynurenine pathway of tryptophan degradation [30], as shown in Figure S3. Additional differentially abundant organic acids may have contributed to differences in tea flavor. Malonic and caffeic acid (upregulated) and tartaric and citric acid (downregulated) have all been reported to have a sour taste [31], and changes in their contents may have influenced the differences in flavor between green tea from the intercropping system and the pure tea garden.

### Carbohydrates

The sugars rhamnose, maltopentaose and D-xylose and the sugar alcohols ribitol, L-iditol and D-threitol were all upregulated in samples under the intercropping vs. monoculture. Rhamnose showed the greatest upregulation (2.7-fold). D-maltopentaose, upregulated 1.30-fold, is an oligosaccharide with low sweetness that is widely used in the food industry and has applications in nutrition and healthcare [32]. D-xylose, a dextrorotary form of xylose, was upregulated 1.49-fold; it is widely used as a diabetic sweetener in foods and beverages and is much sweeter than D-glucose [33], suggesting that it may have a role in the improvement of tea taste.

Sugar alcohols were also increased by various amounts in samples under the intercropping vs. monoculture, and the most strongly upregulated was D-threitol (2.21-fold). Ribitol, also called adonitol, was upregulated 1.33-fold; it is formed by the reduction of ribose in certain plants and contributes to the structure of riboflavin [34]. L-iditol, upregulated 1.40-fold, is a hexitol with a sweet taste that has applications in the food industry. Most sugar alcohols are less sweet than sucrose. However, they also have less food energy, which is particularly important for human health in the present era of excess food and calories [35, 36].

Overall, increased carbohydrates with varying degrees of sweetness and different tastes may have provided raw materials to make green tea brewed from the intercropping samples sweeter and richer, with a better taste.

### Lipids

Jasmine lactone, oleic acid and stearoyl carnitine were upregulated in samples under the intercropping vs. monoculture, and only dodecanoic acid was downregulated. Jasmine lactone and oleic acid upregulated 1.21-fold and 1.68-fold, respectively.

### Flavonoids

Flavonoids were detected only in the positive mode; two were significantly upregulated and four were significantly downregulated. Apiin increased 1.76-fold, whereas apigenin decreased 1.16-fold. Apiin (apigenin 7-O-apioglucoside) is a water-soluble diglycoside of the more hydrophobic apigenin [37], and tea from the intercropping may provide more water-soluble apiin when consumed. More importantly, apiin has no taste, whereas apigenin is bitter: increased apiin and decreased apigenin should therefore reduce the bitterness of brewed tea, increasing its palatability. Additional downregulated flavonoids included naringin and prunasin. Luteolin was upregulated 1.53-fold in samples under the intercropping vs. monoculture. Taken together, our results indicated that the intercropping samples vs. T had lower amounts of the bitter compounds apigenin, naringin and prunasin and greater amounts of the tasteless compound apiin, consistent with the better taste of their tea.

### Secondary metabolite pathway analysis

**Fig. 6:**
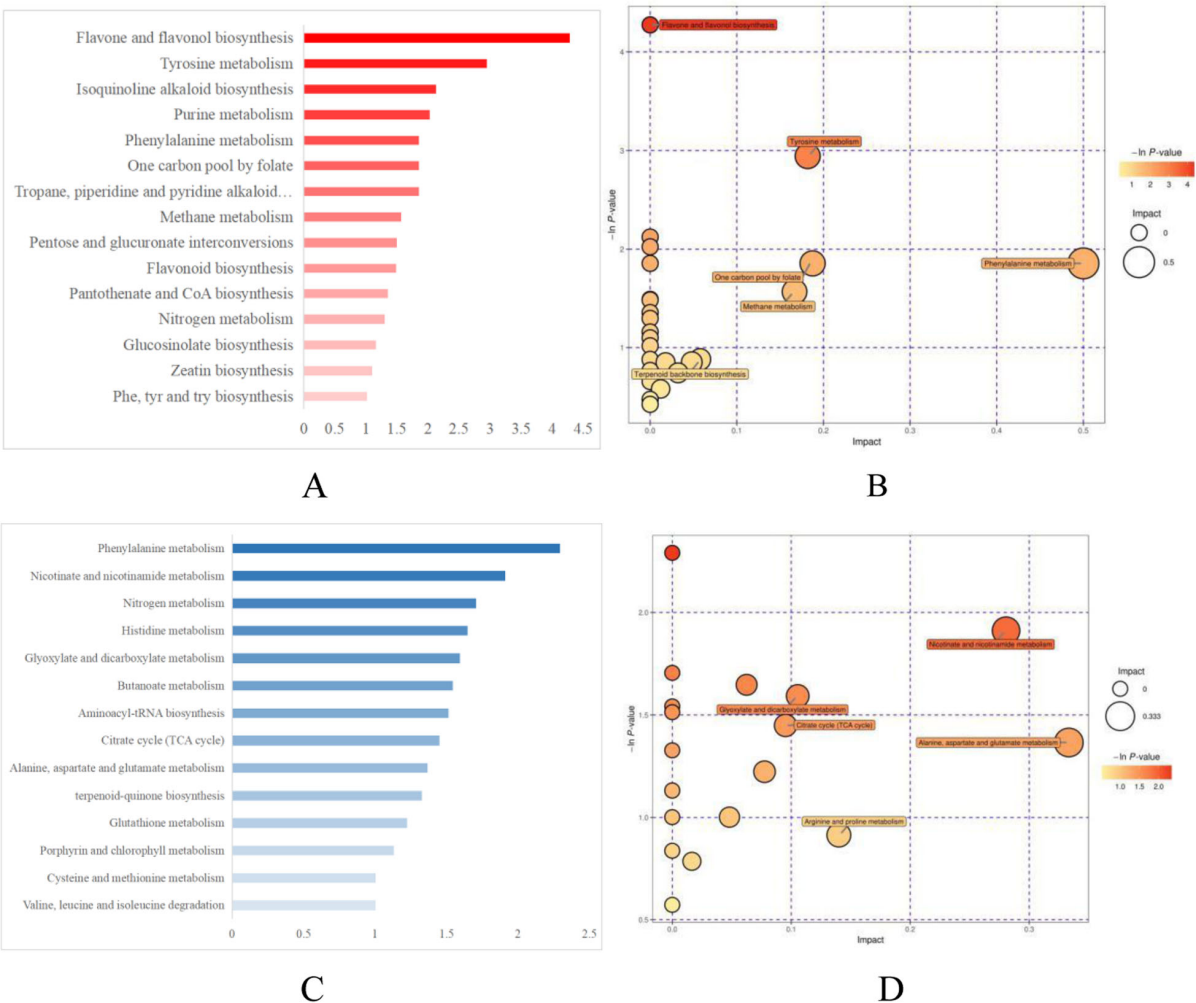
Metabolic or biosynthetic pathways in the positive (**a**, **b**) and negative (**c**, **d**) modes in 80 T (intercropping) vs. T (monoculture) (−ln(P) > 1)

The differentially abundant metabolites mapping to the KEGG database displaying in Fig. 6, 15 metabolic or biosynthetic pathways (Fig. 6a, b) showed differences in positive mode and 14 (Fig. 6c, d) in negative mode for samples under the intercropping vs. monoculture (−ln(P) > 1).

In positive mode, the flavone and flavonol biosynthesis pathway showed the greatest degree of alteration. Upregulated apiin and luteolin and downregulated apigenin are involved in this biosynthetic pathway, and apiin and luteolin are located downstream of apigenin, suggesting that the enzymes of apiin and luteolin biosynthesis were activated in the intercropping system. Luteolin and apigenin are also involved in flavonoid biosynthesis. In addition, naringin was also downregulated. In addition, Val and Phe appeared to have important roles: together they participated in four pathways, including cyanoamino acid metabolism, aminoacyl-tRNA biosynthesis, 2-oxocarboxylic acid metabolism, and biosynthesis of amino acids. Sugars and sugar alcohols were mainly involved in pentose and glucuronate interconversions and ABC transporters.

In negative mode, the Phe metabolism pathway showed the largest difference between intercropping and monoculture. Plants synthesize types of volatile organic compounds to interact with the surrounding environment via Phe metabolism [38]. The products of this pathway are not immediately needed for cell survival but can increase the fitness of the plant as a whole. In our study, eight differentially abundant metabolites were involved in Phe metabolism. Phe itself took part not only in phenylalanine metabolism but also in four other pathways, highlighting its multiple functions and versatility. Some Phe-related metabolites were also conducive to the formation of fragrance: phenylpyruvic acid has a slight honey-like odor, trans-cinnamic acid has a light cinnamon aroma, and phenylacetaldehyde has a strong fruity, nut-like, floral and sweet scent. Together, these metabolites provide abundant raw materials for the production of green tea aromas. Finally, phenylethylamine is a neurotransmitter that raises the level of dopamine in extracellular fluids, making people feel more positive and satisfied; its presence could explain why tea drinking can improve the mood to some extent.

Glu and His appeared together in the four of the top 10 differentially expressed pathways identified in the negative mode, suggesting that they have multiple functions. Glu participated individually in alanine, aspartate and glutamate metabolism; carbon metabolism; 2-oxocarboxylic acid metabolism; and glyoxylate and dicarboxylate metabolism. Likewise, His participated individually in beta-alanine metabolism.

Five pathways showed significant differences in both the positive and negative modes: biosynthesis of amino acids, aminoacyl-tRNA biosynthesis, carbon metabolism, 2-oxocarboxylic acid metabolism, and ABC transporters. The numbers of pathways associated with amino acid metabolism in both the positive and negative modes were remarkably high: five pathways (biosynthesis of amino acids; arginine and proline metabolism; glycine, serine and threonine metabolism; tyrosine metabolism; and tryptophan metabolism) were defined in two modes, and four pathways (biosynthesis of amino acids; alanine, aspartate and glutamate metabolism; histidine metabolism; and beta-alanine metabolism) were identified in the negative mode only. This result suggests that the intercropping system significantly affected amino acid metabolism and thereby altered the accumulation and degradation of amino acids. Combined with the results of the targeted amino acid metabolomics, these results indicated that amino acids with a good taste accumulated in leaves under intercropping compared with monoculture, whereas flavorless amino acids were less abundant.

## Discussion

### Up-regulated delicious amino acids may be the raw material of delicious green tea, while the down-regulated are less responsive to the flavor

Free amino acids contribute to the taste of tea, and taste intensity increases with increasing amino acid concentration [39, 40]. Twenty-nine amino acids were identified in green tea extracts in previous studies, and 11 Amino acids are responsible for the quality of tea infusion and antioxidant and anti-inflammatory functions [12, 41].

Combinations of amino acids with their individual special tastes could expand the possibilities for new tea tastes or functions. For instance, Asp has a sour, slightly umami taste, whereas Phe is tasted a little bitter. Whereas, aspartame which is a conjugate of these two amino acids, is 200 times sweeter than sugar [42]. The simultaneous increase of Asp and Phe could provide a basis for greater aspartame production. Together, our results suggest that upregulation of multiple sweet, umami and floral amino acids in the intercropping samlpes may be the proximate cause of its excellent flavor in green tea.

The bitter-tasting amino acids arginine (Arg), Val, lysine (Lys), tryptophan (Trp), tyrosine (Tyr), and His were also differentially abundant between samples under the intercropping vs. monoculture. These six bitter amino acids can be further classified into two subcategories: Arg, Val, and Lys contribute both bitter and sweet flavors, whereas Tyr, Trp and His are neurotransmitter precursors [43–45]. Due to their important biological activities, the boosts in the contents of these amino acids from intercropping system may increase the health benefits of the resulting tea. Such benefits are another aspect of tea quality.

MHis and 3-MHis are derived primarily from anserine (β-alanyl-1-methyl-histidine) and balenine (β-alanyl-3-methyl-histidine), respectively, and both anserine and balenine have antioxidant functions [46]. The downregulation of 1-MHis and 3-MHis in the intercropping system may have reflected a reduced ability of anserine and balenine to form 1-MHis and 3-MHis, thereby permitting more anserine and balenine to accumulate and perform antioxidant functions. Antioxidants can protect cells by eliminating reactive oxygen species (ROS) [47]. They can also scavenge free radicals in humans if taken in food to treat diseases and protect health [48]. The potential accumulation of anserine and balenine in intercropping tea plants requires further experimental investigation and may provide new evidence for the antioxidant effects of tea.

### Up-regulated GABA may be responsive to the increased bioactivity of green tea

GABA is an important inhibitory neurotransmitter of the central nervous system and has multiple functions in neurology; altered concentrations of GABA in human brain may be associated with various neurological disorders [49]. GABA-enriched foods have been positively developed recently because of their health functions. All kinds of GABA-enriched foods, including GABA tea, are under exploration and production, and it has been reported that intake of GABA-enriched foods can help to prevent from diabetes and control or inhibit cancer cells [50]. The GABA tea has been delivered as a commercial product for people with high blood pressure in Japan. GABA content is higher in fresh tea leaves from intercropping system, suggesting that this production system may be useful for the exploration and production of GABA-enriched tea.

### Organic acids may contribute to the increase of delicious flavors and the neurotransmitter

Allantoate was reported to be vital for translocating N form and metabolic N currency in *Acer* species, and it is the major N form in spring sugar maple sap [51]. The partial intricate maple flavor originates from the Maillard reaction that takes place during the heating process in which allantoin and/or allantoate react with reducing sugars present in the sap [52]. In tea, allantoate may be involved in N transport and metabolism and may influence subsequent tea processing by the Maillard reaction, improving tea flavor in the intercropping samples.

The XA synthesis pathway is thought to be a partial detoxification process that lessens the content of 3-hydroxykynurenine whose spontaneous oxidation causes free radical formation and apoptosis [53]. XA affects multiple molecular targets and signaling systems, and it influences brain function and neurotransmission in a wide range as one of the neuroactive kynurenine metabolites [54]. Moreover, XA can reduce oxidative DNA damage [55]. Nonetheless, there are very few reports on XA in higher plants in general or tea plants in particular, despite the fact that KA is found in tea [56]. This is probably the first report of XA in tea from an intercropping system.

Likewise, the upregulated metabolite 2-furoic acid has also been reported to improve the flavor and freshness in food industry [57]. The organic acid profile of tea changes when the fresh tea leaves are steamed during the manufacturing process: some are degraded, some are esterified, and some are increased to varying degrees [58]. Therefore, the organic acids present in fresh leaves provide the raw material for the final tea flavor and influence the formation of flavor substances during processing, in addition to simply contributing to their own sour taste. Organic acids in green tea can also inhibit bitterness and delay the perception of sweetness, and citric acid in particular can preserve the taste of tea [59]. The alterant organic acids here may be responsible for the improved green tea flavor from the intercropping system, although some of them were present in extremely low quantities and have rarely been reported in tea leaves.

### Up-regulated carbohydrates all contributed to the sweetness

Mono- and oligosaccharides and their corresponding sugar alcohols are sweet, with a few exceptions [60]. Although the most important sweetener is saccharose (sucrose), other sugars and sugar alcohols are also important, differing in sweetness quality and taste intensity and providing different levels of sweetness [61]. The presence of free sugar in tea is also vital for the catechin synthesis, helps soluble solids, and promotes the biosynthesis of flavor components during processing [62]. Thus, the upregulation of carbohydrates is likely to improve the taste of brewed green tea.

Rhamnose, is widespread from bacteria to higher plants [63]; its sweetness is 33% that of sucrose, and it can be used as a sweetener [61]. When both rhamnose and glucose are lost, and the taste is abolished [64], a result that highlights the important role of rhamnose in the taste of tea, despite its relatively low sweetness.

### Jasmine lactone contributes to the fruity, sweetness, floral aroma and oleic acid to health benefits

Jasmine lactone is a lactone aroma component with a strong flavor with peach and apricot which occurs naturally in jasmine oil, gardenia, lily, tea, and other plants and contributes to the fruity, sweet floral aroma of tea [65]. Jasmine lactone is one of the important aroma constituents in tea [66], and the upregulation of jasmine lactone may help to explain the strong aroma of green tea from the intercropping samples. Oleic acid is a C18:1 monounsaturated fatty acid, and fresh tea leaves are rich in oleic acid, which can be broken down during tea processing [67]. Oleic acid has many beneficial effects on cellular health, heart health, weight management, brain function and type 2 diabetes [68]. Oleic acid may therefore contribute health benefits and characteristic flavor compounds during tea manufacturing, improving green tea quality in the intercropping system.

### Flavonoids may result in a decrease of bitterness and increased solubility in the green tea under intercropping

Naringin is a common plant flavonoid glycoside that contributes a bitter taste to tea [69]. Naringenin, the aglycone of naringin, is oxidized to apigenin by flavanone synthase [70]. The cyanogenic glucoside prunasin is the precursor to amygdalin and is also responsible for a very bitter taste in plants [71]. The flavonoid profile of intercropping samples was consistent with decreased bitterness and increased flavonoid solubility, which may have improved both the taste and the bioactivity of green tea.

Luteolin supplies specific anti-inflammatory and anti-carcinogenic effects and controls the development of cancer cells [72]. The interaction of luteolin with cancer cells is similar to that of EGCG, which is a green tea polyphenol inhibiting tumor [73]. Moreover, apiin is also reported to display the functions of anti-inflammatory and anti-cancer [74]. In fact, many flavonoids function as free radical scavengers to protect humans from free radicals [75].

Pathways associated with flavonoids included numerous secondary metabolites with different metabolic functions in plants, including pigment synthesis, stress response and pathogen defense, among others [76], all of which are very important in tea plants. Significant changes in these pathways were also found in previous studies of tea leaves affected by shade treatment [77].

## Conclusion

Green tea brewed from plants grown in a Chinese chestnut–tea intercropping system established in the 1980s on Baohong Mountain (80 T) ranked highest in sensory evaluation and had the lowest ratio of tea polyphenols to amino acids. LC-QTOFMS-based non-targeted metabolomics, UHPLC-MS/MS targeted metabolomics, and multivariate analysis was combined to investigate differences in the metabolite profiles of fresh tea leaves from the intercropping system compared with leaves grown in a pure tea cultivation system (T). We identified 100 differentially abundant metabolites, containing amino acids, organic acids, lipids, carbohydrates, as well as flavonoids. Many were related to flavor and bioactivity and appeared to provide rich raw materials for the improvement of green tea quality, including better taste and greater health benefits. Two pathways that are most closely related to flavor and bioactivity—flavone and flavonol biosynthesis and phenylalanine metabolism—showed the greatest differences in the positive and negative modes, respectively. Tea leaves from the two cropping systems showed numerous differences in pathways related to amino acid metabolism, indicating that the intercropping system greatly influenced amino acid metabolism, altering the accumulation and degradation of amino acid. These results enhance our understanding of the metabolic mechanisms that tea quality is improved in the Chinese chestnut–tea intercropping system, which is conducive to the scientific promotion of the intercropping system of Chinese chestnut and tea in the current situation of increasing shortage of land resources. As for the cause of altered metabolite levels, it may include various factors, including the shade provided by the trees, potential allelopathic effects between the trees and tea plants, below ground interactions between their roots, accumulation of organic material derived from the deciduous trees, etc., all of which require further investigation in the future.

## Supplementary Information

**Additional file 1: Figure S1.** The total ion flow of positive (A) and negative (B) ion modes detected by LC-MS in the tea samples of 80 T (intercropping) vs. T (monoculture). **Figure S2.** Boxplots of absolute quantification of randomly selected amino acids in 80 T (intercropping) vs. T (monoculture). Each box contains the amino acid concentrations from samples of 80 T vs. T. **Figure S3.** Schematic diagram for tryptophan degrading into KA and XA. **Table S1.** Basic information of the tea samples. **Table S2.** Sensory evaluation of green tea samples

## Data Availability

All data generated or analysed during this study are included in this published article and its supplementary information files. The datasets used and/or analysed during the current study are available from the corresponding author on reasonable request.

## Abbreviations

ANOVA: Analysis of variance
BPI: Base peak intensity
CE: Collision energy
ISVF: Ion spray voltage floating
KEGG: Kyoto encyclopedia of genes and genomes
LC-MS: Liquid chromatography coupled with mass spectrometry
m/z: mass-to-charge ratio
MS: Mass spectrometry
MS2: Second mass spectrometer
OPLS-DA: Orthogonal projections to latent structures-discriminate analysis
PCA: Principal component analysis
QC: Quality control
QTOF: Quadrupole time-of-flight
ROS: Reactive oxygen species
RT: Retention time
UHPLC: Ultra high performance liquid chromatography
VIP: Variable importance in the projection
