# Supplementary material for: Non-targeted and targeted metabolomics profiling of tea plants (Camellia sinensis) in response to its intercropping with Chinese chestnut
Source: BMC Plant Biol. 2021 Jan 21;21:55. doi: 10.1186/s12870-021-02841-w (PMC7818752; doi:10.1186/s12870-021-02841-w)
Supplement: Supplementary file 1 — Additional file 1: Figure S1. The total ion flow of positive (A) and negative (B) ion modes detected by LC-MS in the tea samples of 80 T (intercropping) vs. T (monoculture). Figure S2. Boxplots of absolute quantification of randomly selected amino acids in 80 T (intercropping) vs. T (monoculture). Each box contains the amino acid concentrations from samples of 80 T vs. T. Figure S3. Schematic diagram for tryptophan degrading into KA and XA. Table S1. Basic information of the tea samples. Table S2. Sensory evaluation of green tea samples [file 12870_2021_2841_MOESM1_ESM.docx]

**Supplementary**

**Fig S**

**Figure S1.**


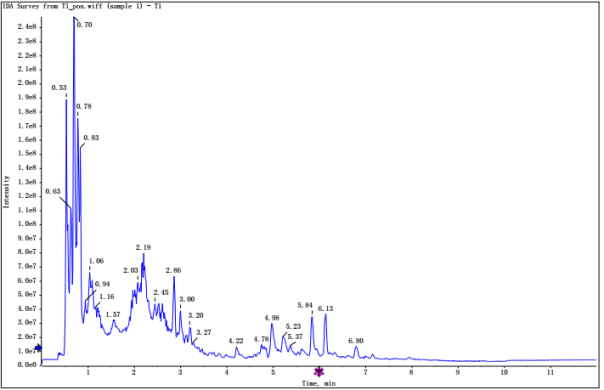

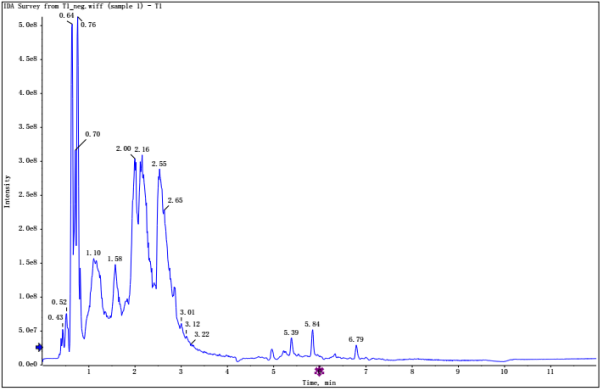


A B

**Figure S2.**

**
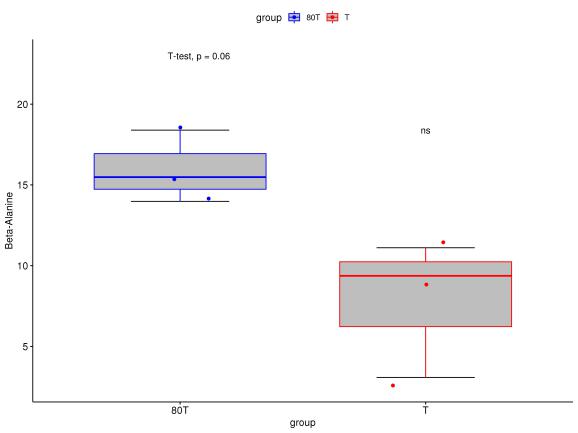

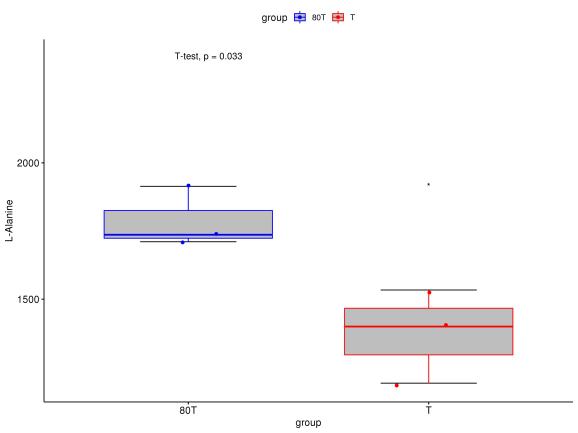

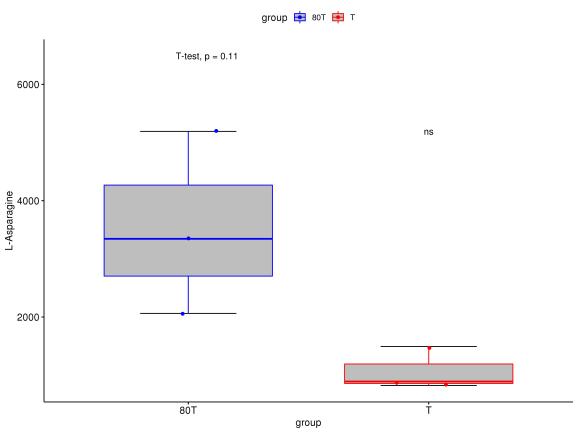

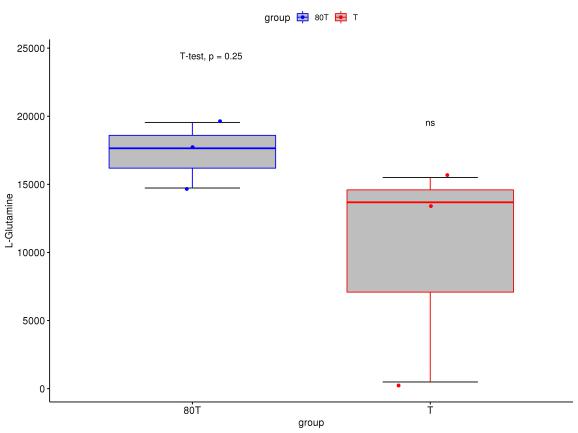

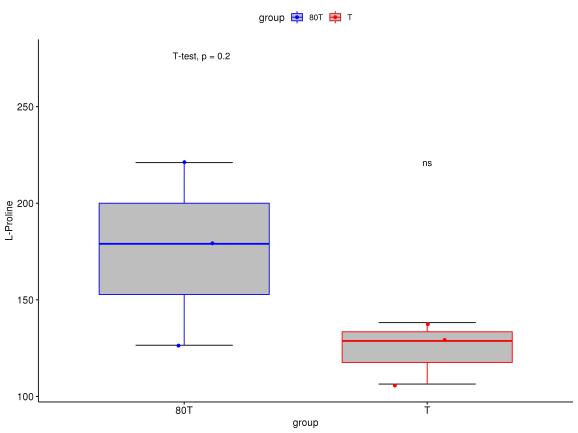

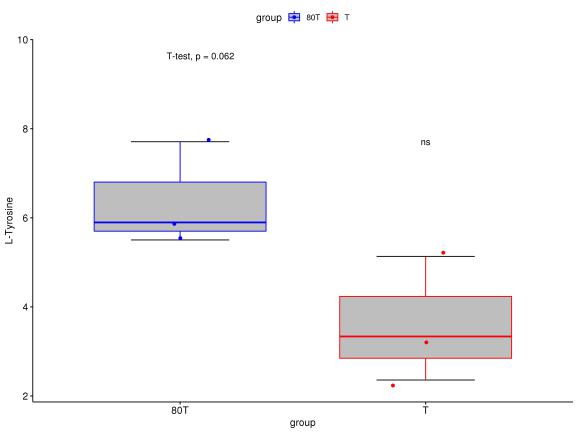
**

**Figure S3.**


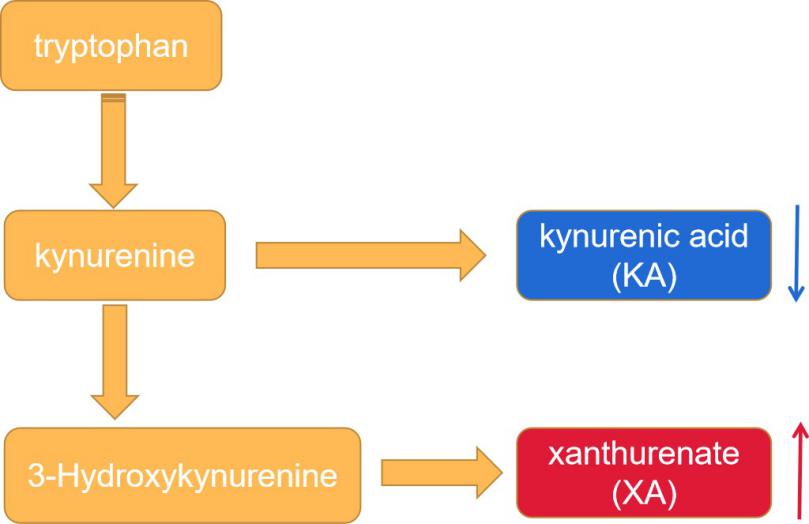


**Table S**

**Table S1.**

| **Description** | **Abbreviation** | **Longitude and Latitude** |
| --- | --- | --- |
| pure tea plantation | T | 103°7′53.91″ 24°59′30.43″ |
| intercropping tea plantation with Chinese chestnut planted in the 1970s | 70T | 103°7′46.48″ 24°59′33.95″ |
| intercropping tea plantation with Chinese chestnut planted in the 1980s | 80T | 103°7′4.01″ 24°59′36.41″ |
| intercropping tea plantation with Chinese chestnut planted in the 1990s | 90T | 103°7′53.94″ 24°59′33.95″ |

**Table S2.**

| **Sample** | **Appearance(20)** | **Color(20)** | **Scent(25)** | **Taste(25)** | **Infused Leaf(10)** | **Gross Score（100）** | **Ranking** |
| --- | --- | --- | --- | --- | --- | --- | --- |
| 70T | 19.5 | 17.5 | 22.5 | 21 | 8 | 88.5 | 2 |
| 80T | 19.5 | 18.5 | 23 | 21.5 | 8.5 | 91 | 1 |
| 90T | 18.5 | 15 | 23 | 18.5 | 6.5 | 81.5 | 4 |
| T | 19 | 16.5 | 20.5 | 19.5 | 7 | 82.5 | 3 |
